# Emergence of potentially disinfection-resistant, naturalized *Escherichia coli* populations across food- and water-associated engineered environments

**DOI:** 10.1038/s41598-024-64241-y

**Published:** 2024-06-12

**Authors:** Daniel Yu, Paul Stothard, Norman F. Neumann

**Affiliations:** 1 School of Public Health, University of Alberta, Edmonton, AB Canada; 2 Antimicrobial Resistance-One Health Consortium, Calgary, AB Canada; 3 Department of Agriculture, Food and Nutritional Sciences, University of Alberta, Edmonton, AB Canada

**Keywords:** Bacterial evolution, Bacterial genomics, Bacterial genetics, Microbial ecology, Food microbiology, Water microbiology

## Abstract

The *Escherichia coli* species is comprised of several ‘ecotypes’ inhabiting a wide range of host and natural environmental niches. Recent studies have suggested that novel naturalized ecotypes have emerged across wastewater treatment plants and meat processing facilities. Phylogenetic and multilocus sequence typing analyses clustered naturalized wastewater and meat plant *E. coli* strains into two main monophyletic clusters corresponding to the ST635 and ST399 sequence types, with several serotypes identified by serotyping, potentially representing distinct lineages that have naturalized across wastewater treatment plants and meat processing facilities. This evidence, taken alongside ecotype prediction analyses that distinguished the naturalized strains from their host-associated counterparts, suggests these strains may collectively represent a novel ecotype that has recently emerged across food- and water-associated engineered environments. Interestingly, pan-genomic analyses revealed that the naturalized strains exhibited an abundance of biofilm formation, defense, and disinfection-related stress resistance genes, but lacked various virulence and colonization genes, indicating that their naturalization has come at the cost of fitness in the original host environment.

## Introduction

*Escherichia coli* is widely recognized as an incredibly diverse species, consisting of a wide range of distinct ‘ecotypes’ that each occupy a specific ecological niche^1^. While *E. coli* typically exists as a benign resident in the vertebrate gastrointestinal tract^2^, select strains have acquired the ability to cause a myriad of intestinal and extraintestinal diseases in human and animal hosts^3,4^. Regardless of its status as a harmless commensal or dangerous pathogen, however, *E. coli* is generally understood to be a host-associated microbe. Indeed, the close association between *E. coli* and its various hosts underscores its use as a prominent fecal indicator bacterium, especially for microbial water quality assessment and fecal source tracking purposes^5^. Despite this, growing evidence points to the existence of distinct ‘naturalized’ populations that have evolved to survive, persist, and even grow in various non-host, natural environments including soil, sediments, and water^6^.

Several studies have differentiated these naturalized populations from their host-associated counterparts, suggesting they represent distinct, environmentally adapted *E. coli* ecotypes. For instance, when compared to host-derived strains naturalized *E. coli* isolated from soil^7,8^ and river water^9,10^ exhibited unique DNA fingerprints based on horizontal fluorophore-enhanced rep-PCR. Similarly, using an accessory gene fingerprinting approach, Tymensen et al.^11^ were able to differentiate naturalized surface water and sediment isolates from enteric strains based on the presence of characteristic combinations of ﻿iron acquisition, complement resistance, and biofilm formation genes. These naturalized populations have also been found to display various phenotypic adaptations reflective of their external, non-host niches. For example, naturalized *E. coli* strains linked with coliform blooms in Australian lakes were found to produce a group 1 capsule, providing enhanced resistance against environmental stressors such as UV radiation and desiccation^12^. Furthermore, *E. coli* strains isolated from temperate and subtropical soils in the United States have been shown to survive long periods of up to 1–2 months at lower temperatures ranging between 4 and 25 °C^7,13^.

Recent evidence suggests that the niche range of *E. coli* may even extend beyond natural environments. Reflecting this, a series of studies conducted by Zhi et al.^14–16^ have described distinct naturalized *E. coli* strains residing in wastewater matrices as a primary niche. Using a novel logic regression-based approach^17^ to intergenic DNA sequence analysis, up to 82% of wastewater *E. coli* isolates were characterized by specific patterns of single nucleotide polymorphisms that were not found in any of their human and animal-derived counterparts^14^. Interestingly, a subset of these wastewater strains also harbored a unique *uspC–*IS30–*flhDC* genetic locus that, at the time, was found to be wastewater-specific^14,16,18^. Reflecting their distinct ecology, the naturalized strains carried an abundance of stress resistance genes, including the transmissible locus of stress tolerance (tLST), that appeared to confer resistance to the extreme stressors (i.e., disinfection) encountered during wastewater treatment^16^. Indeed, when compared to enteric strains, the naturalized wastewater strains displayed an enhanced capacity for biofilm formation^15^, and were found to be resistant to various disinfection-related stressors, including extreme heat, chlorine, and advanced oxidants^19^.

The distinct genotypic, phenotypic, and ecotypic characteristics of these wastewater strains were originally thought to reflect the emergence of a novel, wastewater-specific *E. coli* ecotype that evolved in response to the development of wastewater treatment and sanitation practices^1,16^. Recently, however, Yang et al.^20^ isolated *E. coli* strains from a meat processing plant exhibiting the wastewater-associated *uspC–*IS30–*flhDC* locus and tLST, suggesting that additional *E. coli* populations have become naturalized in other engineered environments. Thus, it is unclear whether these wastewater and meat plant *E. coli* strains represent distinct niche-specific ecotypes or reflect, more broadly, a general naturalized *E. coli* ecotype dispersed across various food- and water-associated built environments. Herein, we demonstrate that these *E. coli* populations can be phylogenetically and ecotypically distinguished from their host-associated counterparts, though they do not appear to represent separate ecotypic groups. Despite this, these wastewater and meat plant groups still harbor specific genetic features reflective of their respective niches, many of which can be linked to resistance against stressors including DNA damaging stimuli (e.g., UV), oxidative stress, heavy metals, and heat shock. Importantly, although these naturalized populations do not appear to be host-associated, their characterization raises the concerning prospect that microbes may be evolving resistance to disinfection.

## Results

### Phylogeny and typing of naturalized wastewater and meat plant *E. coli* strains

**Figure 1:**
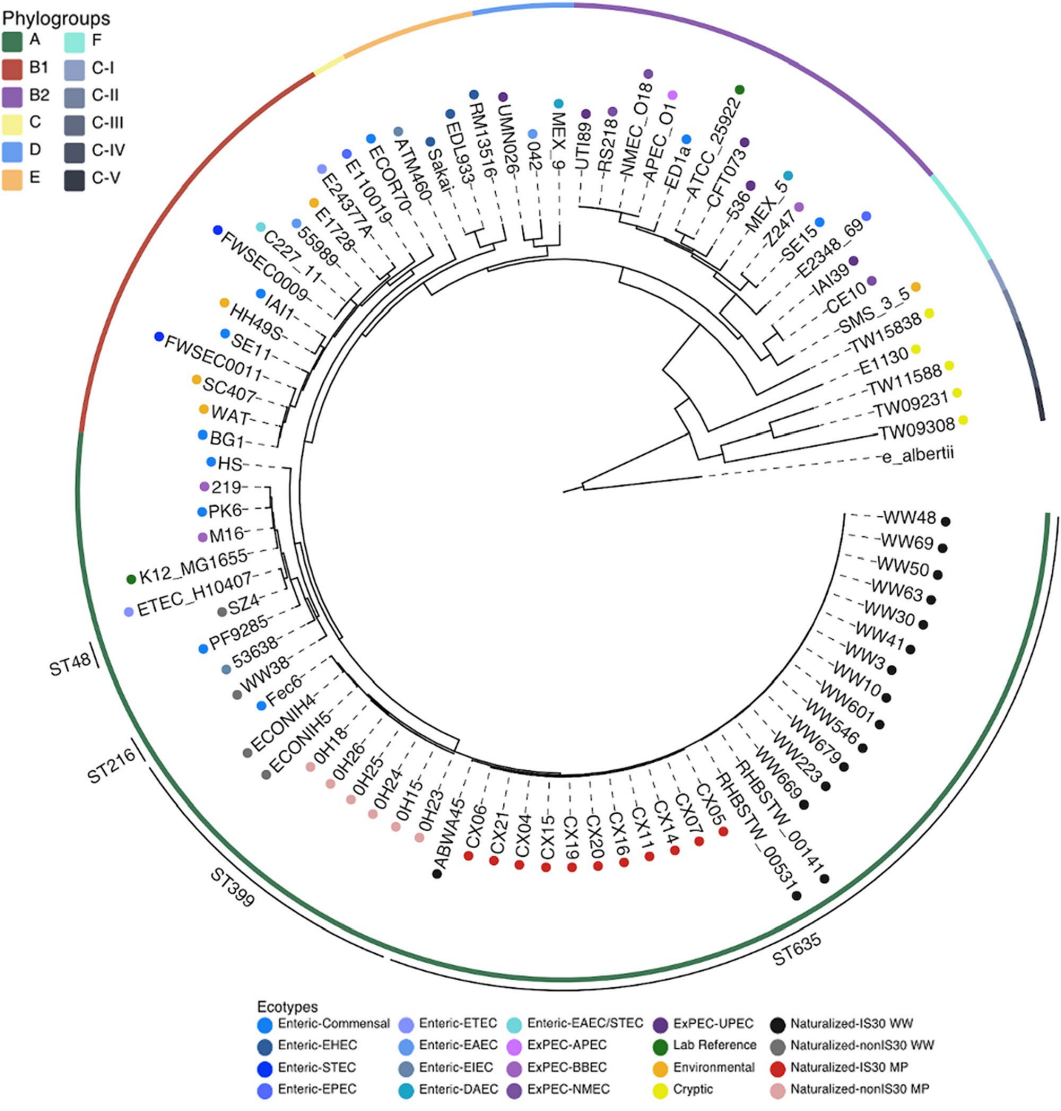
Core genome maximum likelihood phylogenetic tree of naturalized wastewater and meat plant strains alongside other strains representative of the *E. coli* species and the cryptic *Escherichia* clades. The genome sequences of presumptive naturalized wastewater and meat plant *E. coli* strains were screened and downloaded from NCBI GenBank. To evaluate the evolutionary history of these strains in the context of the greater *E. coli* species, the core genome sequence variation of the naturalized wastewater (black and grey circles corresponding to those possessing and lacking the uspC–IS30–flhDC biomarker, respectively) and meat plant (red and pink circles corresponding to those possessing and lacking the uspC–IS30–flhDC biomarker, respectively) strains were compared to enteric (blue circles), ExPEC (purple circles), lab reference *E. coli* (green circles), environmental *E. coli* (orange circles), and cryptic *Escherichia* (yellow circles) strains. Phylogroups of all strains in the phylogenetic tree are indicated by the inner ring and colored according to the upper legend. The main sequence types represented across the naturalized strains are indicated in the outermost ring. The tree is rooted against an *E. albertii* strain as the outgroup.

Alongside a lone naturalized wastewater strain (SZ4) that was isolated and sequenced separately, the genome sequences of 19 naturalized wastewater and 17 naturalized meat plant *E. coli* strains were screened and downloaded from the NCBI GenBank database (see Supplementary Table S1 online). Of these, 16 wastewater strains and 11 meat plant strains were found to harbor the *uspC–*IS30–*flhDC* locus. To assess whether the naturalization of the wastewater and meat plant strains within their respective engineered niches could be reflected in phylogeny, a core-genome phylogenetic analysis was performed. A phylogenetic tree was generated using the maximum likelihood algorithm with the 37 naturalized strains alongside 45 representative *E. coli* strains across lifestyles (i.e., commensal, intestinal pathogenic [InPEC], extraintestinal pathogenic [ExPEC], environmental) and phylogroups, 5 genus *Escherichia* (i.e., non-*E. coli*) strains across the cryptic clades^21^, and an *Escherichia albertii* strain as the outgroup. All naturalized wastewater and meat plant strains were found to cluster within phylogroup A (Fig. 1), with most grouping within a monophyletic clade separate from their host-associated counterparts. Aside from the wastewater strains SZ4 and WW38, which grouped closest to the InPEC strains ETEC_H101407 and 53,638 respectively, the rest of the wastewater and meat plant strains formed a separate cluster that was largely exclusive to the naturalized strains except for the inclusion of Fec6, a presumptive human commensal isolate recovered from a fecal swab sample (Fig. 1).

Despite clustering exclusively within a single phylogroup, the naturalized strains were distributed across multiple sequence types (STs). Twenty-seven naturalized strains were designated as ST635, followed by 8 that were designated as ST399, and one wastewater strain each designated as ST216 and ST48, respectively (Table 1). Interestingly, the wastewater and meat plant strains did not sub-structure according to their original source of isolation, but rather segregated according to the presence of the *uspC–*IS30–*flhDC* locus. Indeed, the ST635 lineage included all naturalized strains carrying the *uspC–*IS30–flhDC locus while the ST399 cluster mainly consisted of naturalized strains that lacked the locus. Serotyping revealed further sub-structuring amongst the naturalized strains, with several serotypes represented across the larger ST clusters identified. For instance, while ST635 contained all wastewater and meat plant strains positive for the *uspC–*IS30–*flhDC* locus, four different serotypes were represented, appearing to coincide with the original geographical source of isolation of the naturalized strains. This included O11:25 for most of the Canadian wastewater strains, O166:H25 for the U.K. wastewater strains, O9/O27:H7 for the lone Swiss wastewater strain, and O10:H25 for the Canadian meat plant strains (Table 1). Similarly, amongst the *uspC–*IS30–*flhDC–*negative naturalized strains comprising the ST399 clade, the rest of the Canadian meat plant strains were assigned the serotype O154:H12, whereas the two U.S. wastewater isolates ECONIH4 and ECONIH5 were designated as O8/O129_13_gp10:H30 and O166:H30, respectively. The two divergent naturalized wastewater strains, SZ4 and WW38, were also found to belong to unique serotypes as they were designated as O64:H20 and O11:H4, respectively. Notably, none of the naturalized strains shared the same serotype with any of the non-naturalized, host-associated strains included in the phylogenetic tree (see Supplementary Table S1 online).

**Table 1:** Distribution of sequence types and serotypes across the naturalized wastewater and meat plant *E. coli* strains.

Strains	Ecotype	Location of isolation	Sequence type	Serotype
WW10, WW223, WW3, WW30, WW41, WW48, WW40, WW546, WW601, WW63, WW669, WW679, WW69	Naturalized wastewater	Canada	ST635	O11:H25
ABWA45		Switzerland		O9/27:H7
RHBSTW_00141, RHBSTW_00531		England		O166:H25
CX04, CX05, CX06, CX07, CX11, CX14, CX15, CX16, CX19, CX20, CX21	Naturalized meat plant	Canada		O10:H25
ECONIH4	Naturalized wastewater	USA	ST399	O18/129_13_gp10:H30
ECONIH5				O166:H30
0H15, 0H18, 0H23, 0H24, 0H25, 0H26	Naturalized meat plant	Canada		O154:H12
WW38	Naturalized wastewater	Canada	ST216	O11:H4
SZ4		China	ST48	O64:H20

### Ecotype prediction with naturalized wastewater and meat plant *E. coli* strains

**Figure 2:**
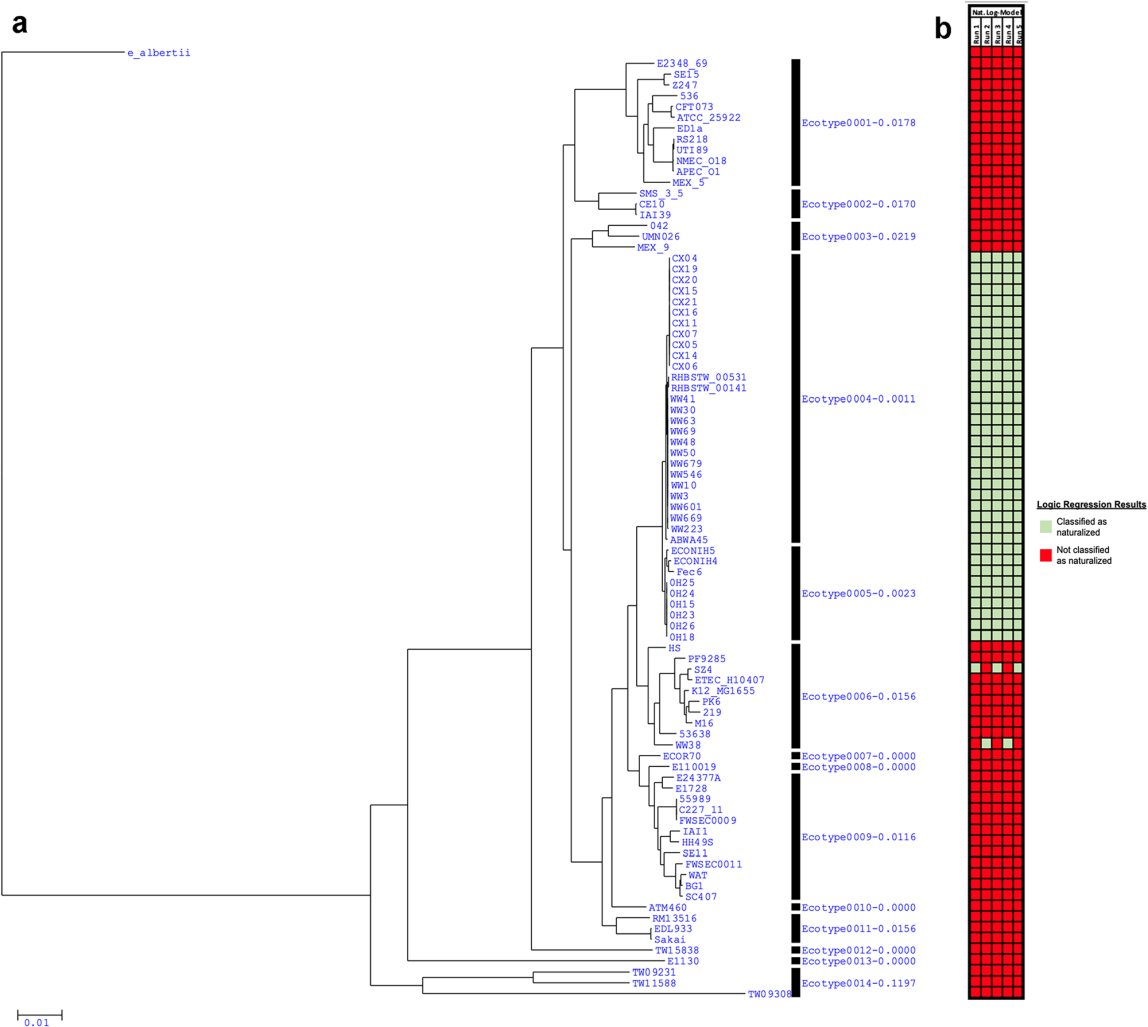
Prediction of putative naturalized *E. coli* ecotypes. Validation of putative naturalized *E. coli* ecotypes using a phylogeny-based (i.e., unsupervised) approach via the Ecotype Simulation 2 algorithm and a supervised learning approach via logic regression. According to the (**a**) phylogeny-based approach, two putative naturalized-associated ecotypes were identified; however, two naturalized wastewater strains, SZ4 and WW38, were not identified as belonging to either of the naturalized ecotypes through this approach. In contrast, across 5 classification trials, the (**b**) logic regression-based approach clustered most naturalized strains into a singular naturalized ecotype, and was also able to correctly classify SZ4 and WW38 on a case-by-case basis. Regardless of the ecotype prediction approach used, however, the enteric strain Fec6 was included in the predicted naturalized ecotype(s) called.

To evaluate whether the phylogenetically distinct naturalized wastewater and meat plant *E. coli* populations could also represent distinct *E. coli* ecotypes, ecotype prediction analyses were performed. Given their distinct phylogenetic placement, a phylogeny-based ecotype prediction algorithm was used first. According to the Ecotype Simulation algorithm, two naturalized-associated ecotypes, designated as ‘Ecotype0004’ and ‘Ecotype0005’, were identified (Fig. 2a). Interestingly, instead of grouping the naturalized strains according to their original ecological niche (i.e., wastewater versus meat plants), ‘Ecotype0004’ was found to correspond to the ST635 naturalized strains harboring the *uspC–*IS30–*flhDC* locus, while ‘Ecotype0005’ included the ST399 naturalized strains and enteric strain Fec6 lacking the locus (Fig. 1). Consequently, the two divergent wastewater strains, SZ4 and WW38, were not assigned to either of the two predicted naturalized ecotypes, but instead clustered into a separate group, ‘Ecotype0006’ (Fig. 2a).

As an alternative method, a supervised learning, logic regression-based approach was also used to cluster the naturalized strains in a phylogeny-independent manner. Based on the sequence variation within the *asnS–ompF* and *csgDEFG–csgBAC* intergenic regions, most wastewater and meat plant strains could be clustered into a single naturalized *E. coli* ecotype despite their differing sources of isolation (Fig. 2b). Across 5 independent classification trials, 35 of the 37 wastewater and meat plant strains were consistently determined to belong to the putative naturalized *E. coli* ecotype. Notably, the ST399 enteric strain Fec6 was also consistently classified as part of the putative naturalized ecotype, whereas the remaining two wastewater strains, SZ4 and WW38, were only classified as naturalized on a trial-by-trial basis, with each being assigned to the naturalized ecotype in 3 and 2 of the 5 classification trials, respectively. Interestingly, the classifications of SZ4 and WW38 appeared to be mutually exclusive, as in any one trial only one of the two strains were classified as naturalized by logic regression.

### Comparative genomics of naturalized wastewater, naturalized meat plant, and host-associated *E. coli* strains

The wastewater and meat plant *E. coli* strains appear to be phylogenetically and ecotypically distinct from other strains in the *E. coli* species—especially when compared to host-derived strains. To further examine the genetic differences between the naturalized populations and their host-associated counterparts, a series of comparative genomic analyses were performed. Starting with average nucleotide identity (ANI), the naturalized strains expectedly shared ≥ 95% ANI with all other *E. coli* strains and the cryptic *Escherichia* clade I strain, but less than 95% ANI with the other cryptic *Escherichia* clade strains (see Supplementary Table S2 online). Consistently, both the naturalized wastewater and meat plant strains were also found to exhibit higher within-group ANI (i.e., amongst other wastewater and/or meat plant strains) than when compared to strains belonging to other ecotypes.

**Figure 3:**
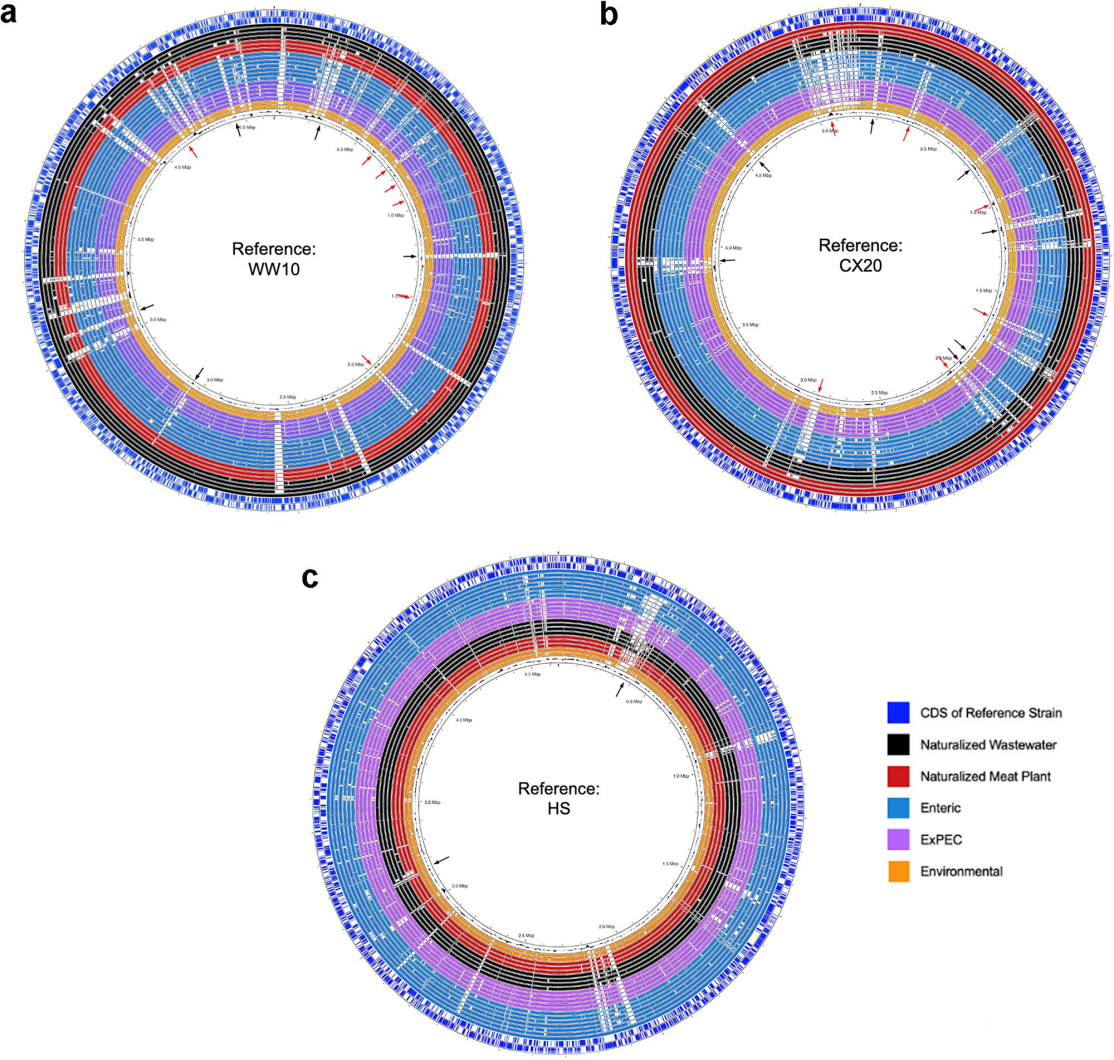
Serial pairwise genomic alignment maps of naturalized wastewater and meat plant strains with enteric, extraintestinal pathogenic, and environmental strains. Genome alignment maps of representative *E. coli* strains belonging to five ecotypic groups, including naturalized wastewater, naturalized meat plant, enteric (including both commensal and InPEC strains), ExPEC, and environmental *E. coli*, rooted against (**a**) a reference naturalized wastewater strain, WW10; (**b**) a reference naturalized meat plant strain, CX20; and (**c**) a reference host-associated strain, HS. In each alignment map, the reference strain's coding sequences (CDS), tRNA genes and rRNA genes are depicted as the outer two blue rings, with the reference genome included as the solid ring just inside the two outer rings. Against each reference genome, strains belonging to different ecotypes are aligned, including, from outermost to innermost: (i) the naturalized wastewater strains (black rings) WW10, ABWA45 and RHBSTW_00141 as uspC–IS30–flhDC–positive strains, and WW38 as a uspC–IS30–flhDC–negative strain; (ii) the naturalized meat plant strains (red rings) CX20 and CX05 as uspC–IS30–flhDC–positive strains and 0H24 as a uspC–IS30–flhDC negative strain; (iii) the enteric strains (blue rings), including HS, Fec6, SE11, SE15, and IAI1 as commensal strains, and EDL933 and E2348_69 as intestinal pathogenic strains; (iv) the ExPEC strains (purple rings) 219, UTI89, CFT073, 536, and CE10; and (v) the environmental strains WAT and SMS_3_5. Gaps in each alignment indicating genetic sequences unique to the reference ecotype (black arrows), as well as those unique to the naturalized ecotype generally (red arrows), are also depicted in the center of the maps.

To assess whether the high within-group genomic similarity shared amongst the wastewater and meat plant strains could correlate with distinct genetic features corresponding to the naturalized lifestyle, pairwise genome alignments were also performed. While all genome maps revealed extensive commonality amongst the strains compared regardless of the reference strain used, several gaps were observed in the alignments. Most gaps were observed in the alignments rooted against the naturalized wastewater (Fig. 3a) and meat plant (Fig. 3b) reference strains, representing genetic regions found in the wastewater and meat plant strains that were absent in their host-associated counterparts. Interestingly, the wastewater- and meat plant-rooted maps also contained regions that were commonly unique or over-represented in both wastewater and meat plant groups, representing genetic regions that were generally characteristic of the naturalized strains. To a lesser extent, gaps were also observed in the map rooted against the host-associated strain (Fig. 3c), representing genetic regions that were missing in the naturalized strains when compared to their host-associated counterparts.

### *Pan*-genome analysis of naturalized wastewater and meat plant E. coli strains against other representative E. coli strains

A pan-genome was estimated for the 37 naturalized wastewater and meat plant strains alongside 76 representative commensal *E. coli*, InPEC, ExPEC, lab reference *E. coli*, environmental *E. coli* and cryptic *Escherichia* strains (see Supplementary Table S1 online). The pan-genome consisted of 37,502 total genes, including 1885 that were ‘core’ and shared by ≥ 98% of the strains included in the analysis (see Supplementary Fig. S1 online). To specifically evaluate the distribution of genes within the estimated pan-genome, a pan-genome spectrum function analysis^22,23^ was performed. In this analysis, the spectrum function produced a curve containing slight internal peaks, suggesting that the genes were non-homogenously distributed across the strains in the pan-genome^24,25^. Interestingly, these internal peaks appeared to coincide with sets of genes that were unique to the naturalized strains (see Supplementary Fig. S2 online).

To identify the specific genes associated with the ecology of the wastewater and meat plant strains, a pan-genome-wide association study was performed. Of the 2501 genes identified by Scoary to be statistically correlated with the naturalized strains, 2082 were found to be over-represented amongst the naturalized strains when compared to other strains in the *E. coli* species, whereas 419 were under-represented (see Supplementary Fig. S3a online). Although 736 genes had no known function, the remaining 1765 were distributed across several functional categories, including those that could be particularly relevant for a naturalized lifestyle (see Supplementary Fig. S3b online). Notably, the naturalized strains appeared to be relatively enriched in genes involved in adhesion and biofilm formation, microbial defense, and stress resistance, but lacked genes associated with virulence and colonization (Fig. 3).

Regarding specific genes important for adhesion and biofilm formation, both wastewater and meat plant strains were found to encode the alternative Yfc fimbrial system which appears to play a role in adhesion to environmental surfaces^26^ (see Supplementary Table S3 online). Beyond this, the wastewater strains encoded components of other alternative fimbrial systems, including Yeh and Elf fimbriae, also involved in environmental adhesion^26^, as well as duplicates of the genes *hns* and *glgS*, which appear to play regulatory roles in biofilm formation. Similarly, the meat plant strains were also characterized by biofilm regulation genes such as *bhsA* and *bigR*, which could enhance control over the formation of biofilms, especially in response to changing environmental conditions.

The naturalized strains were also found to be enriched in genes associated with microbial defense mechanisms. For instance, the naturalized strains harbored an abundance of toxin-antitoxin system genes, including those shared between the wastewater and meat plant strains such as *higAB*, *vapC*, *rcbA*, and *ykfI*, as well as genes specifically associated with the wastewater strains, such as *parDE*, and meat plant strains, including *pemIK, ccdAB*, *yafW*, and *ldrD*, respectively (see Supplementary Table S3 online). Furthermore, the naturalized strains possessed a myriad of defense genes against phages and other invasive mobile genetic elements, including the common anti-phage defense protein *pld*; the meat plant-associated restriction-modification system protein *hsdM*; and the wastewater-associated restriction-modification system proteins *hsdR*, *hindIIIM*, and *hindIIIR,* and the CRISPR-Cas system protein *casC*.

Most notably, the naturalized strains were characterized by an abundance of stress resistance genes. Both wastewater and meat plant strains were enriched in DNA repair and SOS response genes such as *recT*, *recF*, *dam*, *lexA*, *rusA, addA*, *yhcG*, *dinI*, *umuD* and *umuC* (see Supplementary Table S3 online). The naturalized strains also possessed various oxidative stress genes, including common antioxidant proteins such as *adhE* and oxidative damage repair chaperones such as *msrA* and *msrB*. Furthermore, the wastewater strains were also found to harbor the redox modulator *alx*, antioxidant proteins such as *yfcG,* oxidative stress proteins including *stiP* and *yceC*, and the electrophilic stress protein *kefC*, whereas the meat plant strains were characterized by the chlorine resistance proteins *nemA* and *nemR.* Interestingly, despite their role in responses against reactive chlorine species, the naturalized strains appeared to lack the chlorine resistance genes *rclR* and *rclA.*

Beyond DNA-damaging stimuli and oxidative stress, the naturalized strains also harbored various heavy metal resistance systems, including the *cus*, *pco*, and *cop* systems involved in copper resistance, as well as the *sil* system involved in silver resistance (see Supplementary Table S3 online). Furthermore, while the wastewater strains possessed the *ars* arsenical resistance system and the *mer* mercuric resistance system, the meat plant strains harbored additional copper resistance genes including *cueR* and *csoR*. The naturalized strains also possessed various genes involved in other stress responses, including several heat shock proteins such as *htpX, clpC, ftsH4*, *hspA*, *pphA* and *clpP*, and the cold shock protein *ves*. Remarkably, beyond these annotated genes, the wastewater and meat plant strains additionally harbored a myriad of hypothetical proteins that, based on sequence homology, appear to further augment functions related to biofilm formation, microbial defense, and stress resistance (see Supplementary Table S3 online).

While the naturalized strains harbored an abundance of genes associated with functions relevant for survival in their engineered niches, they also appeared to lack genes likely required for success in the original host environment. For instance, the naturalized strains were found to lack various virulence factors, including the ExPEC-associated *kps* capsule biosynthesis genes^27^, various secretion system structural and effector proteins, and a myriad of sequestration proteins involved in the acquisition of iron and other essential metals (see Supplementary Table S3 online). Furthermore, the naturalized strains were also found to lack various host colonization genes, including stress resistance and detoxification genes required for survival during passage through the stomach^28^ and within the gastrointestinal tract^29^, such as *gadA*, *ecdB*, and *vdcD*, as well key colonization factors^30,31^ such as *nanS* and the *ecp* operon (*ecpRABCDE*).

The characteristic over- and under-representation of certain functions within the wastewater and meat plant strains (Fig. 3) suggests that their naturalization could have reduced their fitness within the original host environment. To assess the gene–gene interactions across the pan-genome, particularly those relevant to the naturalized strains, gene association and dissociation networks were generated. Generally, associative gene–gene interactions reflected ecology, as the networks of genes that co-occurred within the naturalized strains were associated with functions relevant for survival within the engineered niche, including biofilm formation, microbial defense, and stress resistance (see Supplementary Fig. S4a online). Conversely, co-occurring genes that were lacking in the naturalized strains were associated with virulence and colonization—functions typically associated with the host environment. Interestingly, interactions between these two groups of genes appeared to be antagonistic as select genes linked with colonization (i.e., ‘*ecpRABCDE*’ and *‘nanS*’) that were under-represented in the naturalized strains formed large dissociation networks with genes involved in stress resistance, biofilm formation, and microbial defense that were conversely over-represented in the naturalized strains (see Supplementary Fig. S4b online).

## Discussion

While *E. coli* is known to colonize a wide range of human and animal hosts, select subpopulations appear to have evolved to reside primarily in natural, non-host environments^6^ as distinct naturalized *E. coli* ‘ecotypes’. In this study, we provide several lines of evidence suggesting that the naturalization phenomenon within *E. coli* has extended beyond natural environments and may include various water and food-associated engineered niches. Given that ecotypes have been suggested to represent the fundamental units of bacterial diversity^32,33^, the prospect that the wastewater and meat plant strains could represent a novel naturalized *E. coli* ecotype was first assessed based on their phylogenetic clustering relative to other strains in the *E. coli* species. Confirming previous analyses^16,19^, all wastewater and meat plant strains clustered within phylogroup A (Fig. 1), a phylogroup that has previously been found to be associated with non-pathogenic strains^34–36^, suggesting that the naturalized strains may be largely non-pathogenic. Despite this, the close grouping of the wastewater strains WW38 and SZ4 with a host-associated counterpart and the inclusion of the enteric strain Fec6 within the near-exclusive naturalized monophyletic cluster (Fig. 1) could suggest that these naturalized populations may have evolved from an originally host-derived strain after repeated passage through wastewater treatment plants and/or meat processing facilities.

Despite their exclusive placement within phylogroup A, the wastewater and meat plant strains were found to sub-cluster into two distinct monophyletic groups within the phylogenetic tree. Interestingly, these monophyletic clusters appeared to correspond closely with two main sequence type lineages, consisting of ST635 strains possessing the *uspC–*IS30–*flhDC* biomarker and ST399 strains without the biomarker, regardless of their original source of isolation. Interestingly, these sequence types have been previously associated with *E. coli* populations recovered from other built environments, particularly those related to water disinfection and sanitation. For instance, septic tank isolates recovered by Behruznia et al.^37,38^ were found to be non-randomly distributed across 3 main lineages, including clonal complex 10, which was proposed to be mainly host- and freshwater-associated, as well as clonal complex 399 and sequence type 401, which were found to be strongly associated with the septic tank niche. Similarly, Constantinides et al.^39^ found that non-clinical *E. coli* isolates colonizing hospital sink drains could be clustered into 4 sequence type lineages, including ST635, ST401, ST472 and ST399. As such, distinct *E. coli* populations, particularly those corresponding to the ST635 and ST399 lineages, appear to be particularly predisposed to becoming naturalized, and specifically within engineered environments associated with food and water sanitation.

The phylogenetic findings raise the prospect that the wastewater and meat plant strains could represent distinct naturalized *E. coli* ecotypes. Reflecting this, two independent ecotype prediction approaches were able to distinguish the naturalized strains from the rest of the *E. coli* species, though the specific ecotypes predicted differed depending on the approach used (Fig. 2). Of the two ecotype prediction approaches used, logic regression appeared to exhibit greater classification power due to its ability to classify a greater number of wastewater and meat plant strains as naturalized. As other studies have utilized a similar method for clustering *E. coli* strains according to their original ecological source with high specificity^16,40,41^, logic regression could represent a novel approach for the identification of putative ecotypes within a bacterial species. Notably, however, regardless of the approach used the wastewater and meat plant strains could not be distinguished based on their original ecological source of isolation. While this could suggest that these two populations collectively represent one general naturalized *E. coli* ecotype dispersed across various engineered environments, this finding could also be due to the current limitations of the ecotype prediction approaches used—especially for logic regression. The logic regression analysis in this study was performed based only on the sequence variation contained within two intergenic regions (i.e., *asnS–ompF* and *csgDEFG–csgBAC*) within the *E. coli* genome. Given that previous analyses have found that different intergenic regions can encode varying degrees of niche-relevant information^41^, the selection of alternative or a greater number of intergenic loci could improve the discrimination power of the logic regression algorithm, and allow for the sub-classification of the wastewater and meat plant strains into distinct ecotypes.

Regardless of the specific ecotypes predicted, the wastewater and meat plant strains appeared to be genetically distinct when compared to other ecotypes in the *E. coli* species. Reflecting this, the wastewater and meat plant strains were found to consistently share higher within-group ANI similarity than when compared to other *E. coli* ecotypic groups (see Supplementary Table S2 online). Although genomic similarity measures like ANI have historically been used for demarcating bacterial species rather than ecotypes^42^, strains that share common ecotypic properties should be more similar genetically than those with different ecotypic traits. As such, the high within-group ANI similarity shared by the naturalized strains provides additional support for their designation as a distinct, naturalized *E. coli* ecotype. Importantly, while the high within-group genomic similarity and monophyletic clustering of the wastewater and meat plant strains indicate that they could all be clonal, the presence of multiple serotypes amongst the naturalized strains (Table 1) suggests that several naturalized *E. coli* lineages have independently emerged across various food- and water-associated engineered environments.

**Figure 4:**
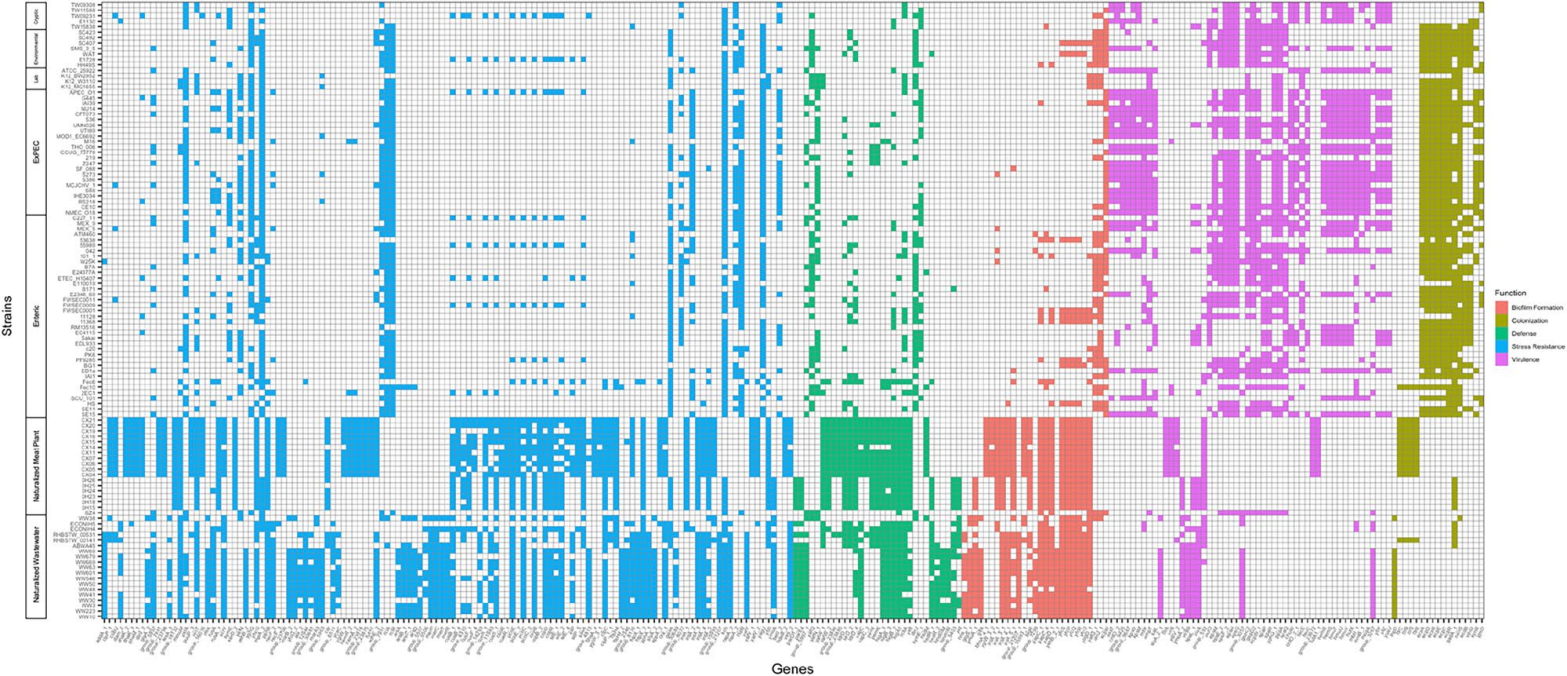
Presence/absence heatmap of genes statistically correlated with the naturalized wastewater and meat plant strains when compared to other strains representative of the *E. coli* species, as determined by Scoary. All genes in the pan-genome estimated by Roary were statistically scored (p < 1E−5, with Benjamini–Hochberg correction) against the naturalized strains to determine genes that could be associated with the distinct ecology of the wastewater and meat plant strains, especially in comparison to strains belonging to the other major *E. coli* ecotypes, including enteric commensal and InPEC strains, ExPEC strains, lab reference strains, environmental (i.e., naturalized in non-engineered environments) strains, and cryptic Escherichia strains. The function of each annotated gene was determined after cross-referencing each gene against their corresponding entry in the UniProt and EcoCyc databases, while the functions of unannotated genes were inferred through sequence homology shared with protein entries in the NCBI Protein database. Several functions were represented across the genes identified by Scoary, and based on the characteristic distribution of genes across the strains analyzed, the wastewater and meat plant strains appeared to be enriched in genes associated with adhesion and biofilm formation (red), microbial defense mechanisms (green) and stress resistance (blue), but were relatively lacking in genes related to colonization (yellow) and virulence (purple).

In line with their designation as a distinct *E. coli* ecotype, the naturalized strains were found to possess distinct genetic features reflecting their adaptation towards the wastewater or meat plant niche. Indeed, the naturalized strains were enriched in various genes associated with biofilm formation, microbial defense, and stress resistance—functions that would presumably be advantageous in non-host contexts (Fig. 4). For instance, the use of alternative fimbrial systems and additional biofilm regulators could enhance the ability of the naturalized strains to form biofilms, thereby increasing their tolerance to the extreme stressors (i.e., low temperatures, disinfection-related stressors [i.e., chlorine, advanced oxidants, UV], etc.) encountered in engineered environments^43–45^. Reflecting this, previous work conducted by Zhi et al*.*^15^ found that naturalized wastewater strains were particularly robust biofilm producers, forming biofilms at roughly three times the capacity of their enteric counterparts. The naturalized strains also harbored an abundance of microbial defense genes, including various toxin-antitoxin systems to survive the intense inter-microbial competition against the complex microbial communities within wastewater matrices^46^ and meat processing facilities^47^. Interestingly, the wastewater strains specifically were found to be enriched in genes associated with restriction-modification and CRISPR-Cas systems, which could protect against the heavy load of phages present in sewage and wastewater^48–50^. Above all else, the naturalized strains were most notably characterized by an over-abundance of stress resistance genes mediating responses against stressors typically encountered during disinfection, including DNA-damaging stimuli (i.e., UV radiation), oxidative stress (i.e., oxidants, reactive oxygen species, reactive electrophilic species, chlorine), heat shock (i.e., composting of human biosolids, steam pasteurization), and heavy metals. Interestingly, a select subset of these stress resistance genes appeared to be niche-specific. For instance, the wastewater strains were found to harbor additional heavy metal resistance systems against arsenic and mercury, heavy metal species that appear to be common constituents in wastewater^51–53^. In contrast, the meat plant strains were found to harbor the chlorine resistance genes *nemA* and *nemR*, which could enhance resistance against the bleach-based sanitizers used in food processing operations. Importantly, reflecting the over-representation of these disinfection-related stress resistance genes within the naturalized groups, the naturalized strains have been found to exhibit enhanced resistance to disinfection related stressors, including against high temperatures up to 60 °C^18,19^, as well as advanced oxidants and chlorine^18^.

The naturalized strains, however, were also found to simultaneously lack various genes likely required for success within the original host environment. The wastewater and meat plant strains, for instance, lacked various metal acquisition and secretion system genes that could enhance fitness within the host environment^54–57^. Furthermore, the naturalized strains also lacked various genes required for the successful colonization of a host gastrotinestinal tract, including for survival during passage through the stomach^28^, key colonization factors^30,31^, and protection against the toxic metabolites produced within the gut^29^. Interestingly, some of these host-colonization genes, such as the *ecpRABCDE* operon and *nanS*, were found to be negatively correlated with the biofilm formation, defense, and stress resistance genes that were over-represented amongst the naturalized strains (see Supplementary Fig. S4b online), suggesting that the genetic adaptations acquired to tolerate the harsh conditions encountered in engineered environments may have come at the cost of fitness within the original host niche (i.e., antagonistic pleiotropy).

Collectively, our evidence points to the existence of distinct naturalized populations of *E. coli* that have evolved to exploit various food- and water-associated engineered environments as primary niches. To date, these naturalized strains have only been isolated from food and water industrial contexts, including meat processing facilities^20^, wastewater^14–16^, septic tanks^37,38^, and sink drains^39^. While the wastewater and meat plant strains assessed in this study do not appear to be pathogenic or even host-associating, their characterization highlights a concerning prospect: that microbes, including those that could represent a pathogenic risk, could be evolving resistance to disinfection. Reflecting this, previous studies have found that repeated exposure to monochloramine water disinfection promoted the development of resistant populations of *E. coli*^58^, with > 60% of cells remaining viable after treatment^59^. Importantly, while this present study focuses on naturalized *E. coli*, the same selective pressures are likely operating for the rest of the microbiome within these engineered environments. For instance, given that many of the genes over-represented in the naturalized strains were duplicates of genes widely distributed across the *E. coli* pan-genome (see Supplementary Table S3 online), other *E. coli* populations could similarly amplify certain key genes to modify their capacity to respond to the extreme stressors (i.e., disinfection) encountered in the engineered environment^60^. Alternatively, these naturalized populations could also act as reservoirs for the dissemination of disinfection resistance to other microbial populations through horizontal gene transfer^61^. Indeed, with studies demonstrating that a significant proportion of *E. coli* strains surviving wastewater treatment are virtually identical to clinical ExPEC across all genomic levels^62,63^, the evolution of disinfection resistance in the microbial world could represent a novel and emerging risk to public health.

## Conclusion

Conventionally, *E. coli* is understood as a host-associated microbe; however, advances in the field of environmental microbiology have led to the characterization of naturalized *E. coli* that have diverged from their host-associated counterparts. This naturalization phenomenon appears to have extended to non-host, non-natural (i.e., man-made) engineered environments as distinct populations of *E. coli*, or ‘ecotypes’, appear to have emerged within wastewater treatment plants and meat processing facilities. Specifically, the phylogenetic, ecotypic, and genomic evidence presented in this study point to the existence of naturalized *E. coli* that have adapted to survive within food- and water-associated engineered environments at a potential cost of fitness in the original host niche. Importantly, even though these naturalized wastewater and meat plant strains no longer appear to be host-associating, the same evolutionary forces underlying their emergence are likely operating for all other microbes present within these environments. Indeed, while they may not represent a direct pathogenic risk to human health, the characterization of these naturalized strains points to a frightening prospect warranting further research—that microbes, including pathogens, could be evolving resistance to disinfection.

## Materials and methods

### Core genome phylogenetics and typing of naturalized strains

All publicly available *E. coli* genomes representing presumptive naturalized wastewater and meat plant strains were identified in the NCBI GenBank database by screening for the *uspC–*IS30–*flhDC* sequence (GenBank Accession Number: ON075843.1) using BLAST. The genome sequences of strains bearing the full *uspC–*IS30–*flhDC* locus were then downloaded from NCBI GenBank (accessed: 01-26-2022) alongside previously-described naturalized wastewater and meat plant strains without the *uspC–*IS30–*flhDC* locus^16,20^. For comparative purposes, the genome sequences of additional representative *E. coli* strains across phylogroups, lifestyles (i.e., commensal, intestinal pathogenic, extraintestinal pathogenic, environmental), and isolation source (i.e., host and environmental niches), as well as *Escherichia* strains across the cryptic clades^21^ (i.e., as an additional environmentally-adapted group of non-*E. coli* strains within the genus *Escherichia*) and an *E. albertii* strain, were also downloaded from NCBI GenBank (accessed: 01-26-2022). The genomes were annotated using Prokka^64^ v1.14.6, after which Roary^65^ v3.13.0 was used to produce a core genome alignment for the generation of a maximum likelihood phylogenetic tree by RAxML^66^ v8.2.12, with *E. albertii* as the outgroup.

The phylogroups of the strains included in the phylogenetic tree were determined through the ClermonTyping method, using the ClermonTyper^67^ v23.06 webserver. Multilocus sequence typing (MLST) was performed using mlst v2.22.0 (https://github.com/tseemann/mlst) with the *Escherichia coli* #1 scheme and cross-referenced against the PubMLST database using the Achtman scheme^68^. The serotypes of each strain were determined with ABRicate (https://github.com/tseemann/abricate) using the EcOH database. The phylogenetic tree was visualized and annotated with R software, using the R packages ggplot2^69^ v3.4.2, ape^70^ v5.4.1, and ggtree^71^ v2.2.4. All information related to the bacterial strains included in the phylogenetic tree can be found in Supplementary Table S1 online.

### Ecotype prediction of naturalized wastewater and meat plant strains

To assess whether the pan-genomic characteristics of the naturalized wastewater and meat plant strains reflect distinct naturalized *E. coli* ecotypes, ecotype prediction analyses were performed. Putative ecotypes represented across the *E. coli* strains analyzed were predicted using two methods. First, a phylogenetics-based approach was employed using the Ecotype Simulation 2 algorithm^72^. To maintain consistency with the analyses described above, the algorithm was run using the core-genome maximum-likelihood phylogenetic tree previously produced by RAxML and the core genome alignment previously produced by Roary, such that all ecotype prediction analyses were performed on the same set of strains used for the phylogenetic analysis.

Additionally, logic regression^17^ was also used as an alternative, phylogeny-independent method to assess whether the naturalized strains could be distinguished as distinct naturalized *E. coli* ecotypes. Following previous workflows^40,41^, the *asnS–ompF* and *csgDEFG–csgBAC* intergenic sequences were screened from all strains included in the analysis using BLAST, extracted using bedtools v2.30.0 (https://github.com/arq5x/bedtools2), and then aligned with Clustal Omega^73^. Using a custom R script (available in supplementary information online S1) the aligned intergenic sequences were analyzed using logic regression to identify key SNP-SNP patterns that could be used to classify the strains as either naturalized or non-naturalized. Five random seed numbers were generated with R such that this classification step could be completed over five separate trials, and the results from each iteration were combined (raw results available in supplementary information S1).

### Genetic similarity of naturalized wastewater and meat plant strains with enteric, extraintestinal pathogenic, and environmental *E. coli*

To assess the degree of genomic similarity shared between and amongst strains belonging to each ecotypic group, an average nucleotide identity (ANI) analysis was performed. Specifically, the ANI shared between each strain was calculated in a pairwise fashion (i.e., between two strains at a time) using fastANI^42^ v1.33 to produce an ANI similarity matrix (see Supplementary Table S2 online). Pairwise whole genome alignments were also performed to identify genetic regions that could be uniquely characteristic to the different ecotypic groups (i.e., naturalized, host-associated, etc.). Strains were selected to represent five *E. coli* ecotypes, including naturalized wastewater (WW10, ABWA45, RHBSTW_00141, WW38), naturalized meat plant (CX20, CX05, 0H24), enteric (HS, Fec6, SE11, SE15, IAI1, EDL933, E2348_69), ExPEC (219, UTI89, CFT073, 536, CE10), and environmental (WAT, SMS_3_5) groups, as indicated in Supplementary Table S1 online. Three sets of pairwise alignments were performed with BLAST, with the genome alignment for each strain rooted against a reference naturalized wastewater strain (*E. coli* WW10), a reference naturalized meat plant strain (*E. coli* CX20), or a reference host-associated strain (*E. coli* HS). The genome alignment maps were then visualized and annotated with the reference strain’s coding sequences (CDS) using the Proksee^74^ webserver.

### Pan-genomic analyses of naturalized wastewater and meat plant strains with enteric, extraintestinal pathogenic, and environmental *E. coli*

A pan-genomic analysis was performed on an expanded repository consisting of the genome sequences of: (a) all naturalized wastewater and meat plant strains; (b) human and animal commensal *E. coli* strains; (c) intestinal pathogenic *E.* coli strains (i.e., EHEC, STEC, EPEC, ETEC, EAEC, EIEC, DAEC); (d) extraintestinal pathogenic *E. coli* strains (i.e., UPEC, BBEC, NMEC, APEC); (e) laboratory reference *E. coli* strains*;* (f) environmental *E. coli* strains (i.e., naturalized in natural contexts); and (g) cryptic *Escherichia* strains (Supplementary Table S1). All strains included in the pan-genomic analysis (n = 113) were annotated using Prokka^64^ v1.14.6, after which a pan-genome was estimated with Roary^65^ v3.13.0. Genes that were left unannotated were screened against all bacterial protein sequences available on the NCBI Protein database with BLAST, and their function was inferred based on sequence homology and the identification of conserved functional domains. The distribution of genes within the estimated pan-genome was then evaluated using a pan-genome spectrum function^22–25^.

To characterize the genomic features underlying the distinct ecology of the naturalized wastewater and meat plant strains, a pan-genome-wide association study (pan-GWAS) was performed. Scoary^75^ v1.6.16 was used to score every gene in the pan-genome to identify genes that were statistically over-represented and under-represented across the naturalized strains. Three separate analyses were performed, to find genes that were correlated with: (a) the naturalized wastewater group specifically; (b) the naturalized meat plant group specifically, and; (c) the naturalized group as a whole. All Scoary runs were performed with the ‘–no_pairwise’ flag and used the Benjamini–Hochberg correction method with a *p*-value cut-off of 1E-5, as recommended by the developers (https://github.com/AdmiralenOla/Scoary).

The results from each run were combined, after which duplicate gene entries, truncated genes, and genes present in fewer than 75% of naturalized strains were screened out. The remaining genes were then broadly classified according to their prevalence amongst the naturalized strains, as either: (a) ‘absent’, if they were not present in any of the naturalized strains; (b) ‘duplicate’, if the gene of interest in the naturalized strains appeared to be a copy of another gene that was already widely prevalent across the strains included in the analysis; (c) ‘shared’, if the gene of interest was the only copy in the naturalized strains, but was still shared amongst other strains in the analysis; (d) ‘unique’, for gene entries that were present only in the naturalized strains, and; (e) ‘variant’, if there were multiple entries for a given gene, but for which specific entries appeared to be particularly over- or under-represented in the naturalized strains. Additionally, the genes were also categorized based on their distribution across the strains analysed, as either: (a) ‘wastewater-dominant’, if the gene entry’s prevalence was 40% higher in the wastewater strains compared to the meat plant strains; (b) ‘meat plant-dominant’, if the gene entry’s prevalence was 40% higher in the meat plant strains compared to the wastewater strains; (c) ‘common across wastewater and meat plant’, if the gene entry exhibited greater than 50% prevalence in both the wastewater and meat plant strains, but with no significant difference in sensitivity between the two groups; and (d) ‘lacking in wastewater and meat plant’, if the gene entry was under-represented in both groups of strains. All genes that were found to be statistically correlated with the naturalized groups by Scoary were functionally annotated after reference to the UniProt^76^ and EcoCyc^77^ databases. The distribution of these genes across the strains analyzed was then visualized through a presence/absence heatmap produced with R software using the ggplot2 v3.4.2 package^69^.

Coinfinder^78^ v1.2.1 was used to evaluate the interactions between accessory genes within the pan-genome and identify important gene association (i.e., the presence of one gene is linked to the presence of another) and dissociation (i.e., one gene is present specifically when another is absent) events occurring within the genomic background of the naturalized strains. Pan-genome association and dissociation networks were produced using Coinfinder based on the estimated pan-genome produced by Roary and a core genome phylogenetic tree produced with FastTree^79^ v2.1.11, with the Bonferroni correction method, as recommended by the developers (https://github.com/fwhelan/coinfinder). Gene–gene interaction network maps were then visualized using the Fruchterman Reingold layout with the Gephi platform^80^ and annotated using Inkscape software.

### Supplementary Information

Supplementary Information 1.

Supplementary Information 2.

## Data Availability

All genomic data for the strains analysed in this study can be publicly accessed from the NCBI GenBank database, with all GenBank accession numbers included in the supplementary materials. All other relevant data required to evaluate the conclusions presented are included in this manuscript and/or are available in the supplementary information.

## References

[1] Yu D, Banting G, Neumann NF (2021). A review of the taxonomy, genetics, and biology of the genus *Escherichia* and the type species *Escherichia coli*. Can. J. Microbiol..

[2] Tenaillon O, Skurnik D, Picard B, Denamur E (2010). The population genetics of commensal *Escherichia coli*. Nat. Rev. Microbiol..

[3] Geurtsen J (2022). Genomics and pathotypes of the many faces of *Escherichia coli*. FEMS Microbiol. Rev..

[4] Denamur E, Clermont O, Bonacorsi S, Gordon D (2021). The population genetics of pathogenic *Escherichia coli*. Nat. Rev. Microbiol..

[5] Holcomb DA, Stewart JR (2020). Microbial indicators of fecal pollution: Recent progress and challenges in assessing water quality. Curr. Environ. Heal. Rep..

[6] Jang J (2017). Environmental *Escherichia coli*: Ecology and public health implications—a review. J. Appl. Microbiol..

[7] Ishii S, Ksoll WB, Hicks RE, Sadowsky MJ (2006). Presence and growth of naturalized *Escherichia coli* in temperate soils from Lake Superior Watersheds. Appl. Environ. Microbiol..

[8] Byappanahalli MN (2012). The population structure of *Escherichia coli* isolated fromsubtropical and temperate soils. Sci. Total Environ..

[9] Jang J (2011). Prevalence of season-specific *Escherichia coli* strains in the Yeongsan River Basin of South Korea. Environ. Microbiol..

[10] Jang J (2015). Dynamic changes in the population structure of *Escherichia coli* in the Yeongsan River basin of South Korea. FEMS Microbiol. Ecol..

[11] Tymensen LD (2015). Comparative accessory gene fingerprinting of surface water *Escherichia coli* reveals genetically diverse naturalized population. J. Appl. Microbiol..

[12] Power ML, Littlefield-Wyer J, Gordon DM, Veal DA, Slade MB (2005). Phenotypic and genotypic characterization of encapsulated *Escherichia coli* isolated from blooms in two Australian lakes. Environ. Microbiol..

[13] Ishii S (2010). Factors controlling long-term survival and growth of naturalized *Escherichia coli* populations in temperate field soils. Microbes Environ..

[14] Zhi S (2016). Evidence of naturalized stress-tolerant strains of *Escherichia coli* in municipal wastewater treatment plants. Appl. Environ. Microbiol..

[15] Zhi, S., Banting, G. S., Ruecker, N. J. & Neumann, N. F. Stress resistance in naturalised waste water *E. coli* strains. *J. Environ. Eng. Sci.* **12**, 42–50 (2017).

[16] Zhi S (2019). Evidence for the evolution, clonal expansion and global dissemination of water treatment-resistant naturalized strains of *Escherichia coli* in wastewater. Water Res..

[17] Ruczinski I, Kooperberg C, Leblanc M (2003). Logic regression. J. Comput. Graph. Stat..

[18] Zhi, S., Banting, G. & Neumann, N. F. Development of a qPCR assay for the detection of naturalized wastewater *E. coli* strains. *J. Water Health* **20**, 727–736 (2022). 10.2166/wh.2022.014 35482388

[19] Wang Z (2020). The locus of heat resistance confers resistance to chlorine and other oxidizing chemicals in *Escherichia coli*. Appl. Environ. Microbiol..

[20] Yang X, Tran F, Zhang P, Wang H (2021). Genomic and phenotypic analysis of heat and sanitizer resistance in *Escherichia coli* from beef in relation to the locus of heat resistance. Appl. Environ. Microbiol..

[21] Walk, S.T. The “Cryptic” *Escherichia*. EcoSal Plus **6**(2). 10.1128/ecosalplus.esp-0002-2015 (2015). 10.1128/ecosalplus.esp-0002-2015 PMC11575852 26435255

[22] Baumdicker F, Hess WR, Pfaffelhuber P (2012). The infinitely many genes model for the distributed genome of bacteria. Genome Biol. Evol..

[23] Collins RE, Higgs PG (2012). Testing the infinitely many genes model for the evolution of the bacterial core genome and pangenome. Mol. Biol. Evol..

[24] Moldovan MA, Gelfand MS (2018). Pangenomic definition of prokaryotic species and the phylogenetic structure of *Prochlorococcus* spp. Front. Microbiol..

[25] Gordienko, E. N., Kazanov, M. D. & Gelfand, M. S. Evolution of pan-genomes of *Escherichia coli*, *Shigella* spp., and *Salmonella* enterica. *J. Bacteriol.* **195**, 2786–2792 (2013). 10.1128/JB.02285-12 PMC3697250 23585535

[26] Korea CG, Badouraly R, Prevost MC, Ghigo JM, Beloin C (2010). *Escherichia coli* K-12 possesses multiple cryptic but functional chaperone-usher fimbriae with distinct surface specificities. Environ. Microbiol..

[27] Sarowska J (2019). Virulence factors, prevalence and potential transmission of extraintestinal pathogenic *Escherichia coli* isolated from different sources: Recent reports. Gut. Pathog..

[28] Tramonti A, De Canio M, Delany I, Scarlato V, De Biase D (2006). Mechanisms of transcription activation exerted by GadX and GadW at the gadA and gadBC gene promoters of the glutamate-based acid resistance system in *Escherichia coli*. J. Bacteriol..

[29] Cueva C (2010). Antimicrobial activity of phenolic acids against commensal, probiotic and pathogenic bacteria. Res. Microbiol..

[30] Steenbergen SM, Jirik JL, Vimr ER (2009). YjhS (NanS) is required for *Escherichia coli* to grow on 9-O-acetylated N-acetylneuraminic acid. J. Bacteriol..

[31] Garnett JA (2012). Structural insights into the biogenesis and biofilm formation by the *Escherichia coli* common pilus. Proc. Natl. Acad. Sci. U. S. A..

[32] Cohan FM, Perry EB (2007). A systematics for discovering the fundamental units of bacterial diversity. Curr. Biol..

[33] Koeppel A (2008). Identifying the fundamental units of bacterial diversity: A paradigm shift to incorporate ecology into bacterial systematics. Proc. Natl. Acad. Sci. U. S. A..

[34] Escobar-Páramo P (2004). A specific genetic background is required for acquisition and expression of virulence factors in *Escherichia coli*. Mol. Biol. Evol..

[35] Li B (2010). Phylogenetic groups and pathogenicity island markers in fecal *Escherichia coli* isolates from asymptomatic humans in china. Appl. Environ. Microbiol..

[36] Hutton TA (2018). Phylogroup and virulence gene association with clinical characteristics of *Escherichia coli* urinary tract infections from dogs and cats. J. Vet. Diagnostic Investig..

[37] Behruznia M, O’Brien CL, Gordon DM (2022). Prevalence, diversity and genetic structure of *Escherichia coli* isolates from septic tanks. Environ. Microbiol. Rep..

[38] Behruznia M, Gordon DM (2022). Molecular and metabolic characteristics of wastewater associated *Escherichia coli* strains. Environ. Microbiol. Rep..

[39] Constantinides, B. *et al.* Genomic surveillance of *Escherichia coli* and *Klebsiella* spp. in hospital sink drains and patients. *Microb. Genomics* **6**, 4–16 (2020). 10.1099/mgen.0.000391 PMC7478627 32553019

[40] Zhi S (2015). Assessing host-specificity of *Escherichia coli* using a supervised learning logic-regression-based analysis of single nucleotide polymorphisms in intergenic regions. Mol. Phylogenet. Evol..

[41] Zhi S (2016). An evaluation of logic regression-based biomarker discovery across multiple intergenic regions for predicting host specificity in *Escherichia coli*. Mol. Phylogenet. Evol..

[42] Jain C, Rodriguez-R LM, Phillippy AM, Konstantinidis KT, Aluru S (2018). High throughput ANI analysis of 90K prokaryotic genomes reveals clear species boundaries. Nat. Commun..

[43] Yin, W., Wang, Y., Liu, L. & He, J. Biofilms: The microbial “protective clothing” in extreme environments. *Int. J. Mol. Sci.* **20**, (2019). 10.3390/ijms20143423 PMC6679078 31336824

[44] Fernández-Gómez, P. *et al.* Biofilm formation ability and tolerance to food-associated stresses among ESBL-producing *Escherichia coli* strains from foods of animal origin and human patients. *LWT - Food Sci. Technol.* **168**, (2022).

[45] Chattopadhyay, I., J, R. B., Usman, T. M. M. & Varjani, S. Exploring the role of microbial biofilm for industrial effluents treatment. *Bioengineered* **13**, 6420–6440 (2022). 10.1080/21655979.2022.2044250 PMC8974063 35227160

[46] Cydzik-Kwiatkowska A, Zielińska M (2016). Bacterial communities in full-scale wastewater treatment systems. World J. Microbiol. Biotechnol..

[47] Zwirzitz, B. *et al.* The sources and transmission routes of microbial populations throughout a meat processing facility. *NPJ Biofilms Microbiomes* **6**, 1–12 (2020). 10.1038/s41522-020-0136-z PMC7351959 32651393

[48] Ballesté E (2022). Bacteriophages in sewage: Abundance, roles, and applications. FEMS Microbes.

[49] Strange JES, Leekitcharoenphon P, Møller FD, Aarestrup FM (2021). Metagenomics analysis of bacteriophages and antimicrobial resistance from global urban sewage. Sci. Rep..

[50] Runa, V., Wenk, J., Bengtsson, S., Jones, B. V. & Lanham, A. B. Bacteriophages in biological wastewater treatment systems: occurrence, characterization, and function. *Front. Microbiol.* **12**, (2021). 10.3389/fmicb.2021.730071 PMC8600467 34803947

[51] Qasem, N. A. A., Mohammed, R. H. & Lawal, D. U. Removal of heavy metal ions from wastewater: a comprehensive and critical review. *NPJ Clean Water* **4**, (2021).

[52] Ungureanu G, Santos S, Boaventura R, Botelho C (2015). Arsenic and antimony in water and wastewater: Overview of removal techniques with special reference to latest advances in adsorption. J. Environ. Manage..

[53] Suess E (2020). Mercury loads and fluxes from wastewater: A nationwide survey in Switzerland. Water Res..

[54] Garcia EC, Brumbaugh AR, Mobley HLT (2011). Redundancy and specificity of *Escherichia coli* iron acquisition systems during urinary tract infection. Infect. Immun..

[55] Ho TD, Davis BM, Ritchie JM, Waldor MK (2008). Type 2 secretion promotes enterohemorrhagic *Escherichia coli* adherence and intestinal colonization. Infect. Immun..

[56] Slater SL, Sågfors AM, Pollard DJ, Ruano-Gallego D, Frankel G (2018). The type III secretion system of pathogenic *Escherichia coli*. Escherichia coli, a Versatile Pathogen.

[57] Serapio-Palacios A (2022). Type VI secretion systems of pathogenic and commensal bacteria mediate niche occupancy in the gut. Cell Rep..

[58] Daer S, Rehmann E, Rehmann J, Ikuma K (2022). Development of resistance in *Escherichia coli* against repeated water disinfection. Front. Environ. Sci..

[59] Daer S, Goodwill JE, Ikuma K (2021). Effect of ferrate and monochloramine disinfection on the physiological and transcriptomic response of *Escherichia coli* at late stationary phase. Water Res..

[60] Kondrashov FA (2012). Gene duplication as a mechanism of genomic adaptation to a changing environment. Proc. R. Soc. B Biol. Sci..

[61] Yu D, Ryu K, Zhi S, Otto SJG, Neumann NF (2022). Naturalized *Escherichia coli* in wastewater and the co-evolution of bacterial resistance to water treatment and antibiotics. Front. Microbiol..

[62] Zhi S (2020). Characterization of water treatment-resistant and multidrug-resistant urinary pathogenic *Escherichia coli* in treated wastewater. Water Res..

[63] Yu, D. *et al.* Differential survival of potentially pathogenic, septicemia- and meningitis-causing *E. coli* across the wastewater treatment train. *NPJ Clean Water* **5**, 1–12 (2022).

[64] Seemann T (2014). Prokka: Rapid prokaryotic genome annotation. Bioinformatics.

[65] Page AJ (2015). Roary: Rapid large-scale prokaryote pan genome analysis. Bioinformatics.

[66] Stamatakis A (2014). RAxML version 8: A tool for phylogenetic analysis and post-analysis of large phylogenies. Bioinformatics.

[67] Beghain J, Bridier-Nahmias A, Nagard HL, Denamur E, Clermont O (2018). ClermonTyping: An easy-to-use and accurate in silico method for *Escherichia* genus strain phylotyping. Microb. Genomics.

[68] Jolley, K. A., Bray, J. E. & Maiden, M. C. J. Open-access bacterial population genomics: BIGSdb software, the PubMLST.org website and their applications. *Wellcome Open Res.* **3**, 1–20 (2018). 10.12688/wellcomeopenres.14826.1 PMC6192448 30345391

[69] Wickham, H. *Elegant Graphics for Data Analysis: ggplot2*. *Applied Spatial Data Analysis with R* (2009).

[70] Paradis, E. & Schliep, K. Ape 5.0: An environment for modern phylogenetics and evolutionary analyses in R. *Bioinformatics* **35**, 526–528 (2019). 10.1093/bioinformatics/bty633 30016406

[71] Yu G, Smith DK, Zhu H, Guan Y, Lam TTY (2017). Ggtree: An R package for visualization and annotation of phylogenetic trees with their covariates and other associated data. Methods Ecol. Evol..

[72] Wood, J. M., Becraft, E. D., Krizanc, D., Cohan, F. M. & Ward, D. M. Ecotype Simulation 2: An improved algorithm for efficiently demarcating microbial species from large sequence datasets. *bioRxiv* 2020.02.10.940734 (2020).

[73] Sievers F (2011). Fast, scalable generation of high-quality protein multiple sequence alignments using Clustal Omega. Mol. Syst. Biol..

[74] Grant JR (2023). Proksee: In-depth characterization and visualization of bacterial genomes. Nucleic Acids Res..

[75] Brynildsrud O, Bohlin J, Scheffer L, Eldholm V (2016). Rapid scoring of genes in microbial pan-genome-wide association studies with Scoary. Genome Biol..

[76] Bateman A (2017). UniProt: The universal protein knowledgebase. Nucleic Acids Res..

[77] Karp PD (2018). The EcoCyc database. EcoSal Plus.

[78] Whelan FJ, Rusilowicz M, McInerney JO (2020). Coinfinder: Detecting significant associations and dissociations in pangenomes. Microb. Genomics.

[79] Price MN, Dehal PS, Arkin AP (2010). FastTree 2—Approximately maximum-likelihood trees for large alignments. PLoS One.

[80] Bastian, M., Heymann, S. & Jacomy, M. Gephi: An Open Source Software for Exploring and Manipulating Networks Visualization and Exploration of Large Graphs. In *Proc. Int. AAAI Conf. Web Soc. Media*, pp. 361–362 (2009).

